# Data-Driven Site Occupancy Statistics in Cubic Prussian Blue

**DOI:** 10.1021/acsphyschemau.5c00001

**Published:** 2025-04-08

**Authors:** Sebastian Baumgart, Axel Groß, Mohsen Sotoudeh

**Affiliations:** † Institute of Theoretical Chemistry, 9189 Ulm University, Oberberghof 7, 89081 Ulm, Germany; ‡ Helmholtz Institute Ulm (HIU) for Electrochemical Energy Storage, Helmholtzstraße 11, 89081 Ulm, Germany

**Keywords:** theoretical study, DFT, battery, cathode material, Prussian Blue, sodium configurations, site occupancy statistics

## Abstract

Sodium-ion batteries are emerging as a cost-effective and sustainable alternative to the lithium-ion technology. Prussian blue compounds are demonstrating considerable potential as cathode materials, offering exceptional structural stability and rapid sodium-ion diffusion capabilities. However, in spite of the importance of Prussian Blue for the emerging technology of sodium-ion batteries, surprisingly many atomistic details of the structural changes upon charging and dis-charging are not yet clarified. This study aims to assess stable sodium configurations and derive reliable site occupancy statistics. We employ periodic density functional theory (DFT) to construct the first complete convex hull for the cubic system, encompassing all 24d sites, thereby exploring the entire configurational space available within these compounds. We identify a new, more stable sodium arrangement within the fully sodiated, cubic Prussian Blue structure, which has to be considered for reliable atomistic modeling. The convex hull identifies a single stable intermediate sodium concentration (*x* = 1), which aligns with observed voltage plateaus in open-circuit voltage measurements. Furthermore, a comparative analysis of the cubic phase and its rhombohedral counterpart is conducted, demonstrating qualitative consistency with phase transition for higher sodium concentrations (*x* > 1). These results strengthen the evidence that Prussian Blue compounds offer exceptional potential as cathode materials, providing valuable insights into their intricate sodium orderings.

## Graphical abstract

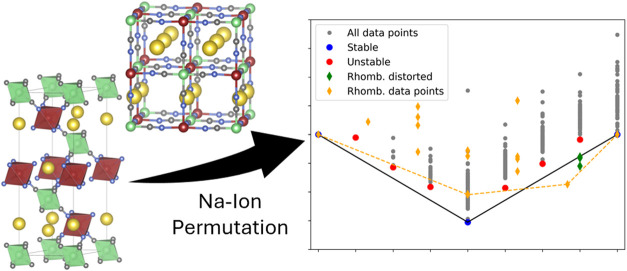

## Introduction

In recent years the sustainability crisis of especially lithium has become increasingly recognized by the public. The opposition to the continued use of lithium in future energy storage is most often justified by the expected resource depletion and concomitant future supply risk.^1^ Additional factors that should be considered include the inhomogeneous distribution of deposits, supply chain risks, the environmental impact of the necessary mining operations, and ethical concerns.^1–4^ To develop alternative energy storage solutions that are not based on critical resources,^5,6^ numerous research institutions and companies have dedicated a significant portion of their research and funding to commercially viable sodium-ion batteries (SIBs). Currently, the three main contenders for the cathode material of such SIBs are layered oxides,^7–9^ polyanionic compounds,^8–11^ and Prussian Blue type materials.^12,13^ Layered oxides are typically formed by layers of edge-sharing MO_6_ octahedra.^14^ Historically, layered oxides were the first cathodes to be used in commercial lithium-ion batteries (LiCoO_2_, LCO^15^) and continue to make up part of the commercialized cathode materials with representatives like Ni–Mn–Co oxide (NMC) or Ni–Co–Al oxide (NCA). In regards to postlithium batteries the research focuses on layered oxide materials with the general formula Na_*x*_MO_2_ (M = Fe, Mn, Ni, Ti, Cr, or Co).^16,17^ All of these are contending for dominance as the best choice for cathode materials for SIBs especially due to their large energy density, high capacity and ease of synthesis.^18^ Polyanionic compounds, such as sodium (NA) Super Ion CONductor (NASICON) compounds or sodium (N) Vanadium Phosphate (NVP) type compounds are materials that contain anions with multiple negative charges, such as the tetrahedral anionic units XO_4_ or any of their derivatives.^19^ They are promising candidates due to their especially high ionic conductivity and strong structural stability.^20^ Prussian Blue (PB, Na_0–2_Fe­[Fe­(CN)_6_]) and its analogues (PBAs, Na_0–2_B­[B′(CN)_6_] where B and B′ are transition metals) are strong contenders for a very sustainable, sodium based battery value chain.^12,13^ While the energy density of these materials is lower compared to lithium-ion batteries or the other contenders presented above, their use will solve a lot of issues regarding the planned massive expansion of grid storage solutions due to their cheap and easy synthesis and the homogeneous global distribution of raw materials.^21,22^ The results of the research are currently starting to show, as large companies like CATL,^23^ Altris AB,^24^ and Natron Energy^21^ prepare to produce their first SIBs. The aforementioned selection of companies share one unique attribute: they all focus on Prussian Blue-based compounds as the cathode material in their upcoming sodium ion battery production.

On the atomic level, the lattice of Prussian Blue is made up of cyanide ligands, which interconnect high-spin, nitrogen coordinated iron atoms with low-spin carbon-coordinated iron atoms in an alternating fashion along all three spatial directions.^25–27^ During the (de)­intercalation of charge carriers, such as sodium, both species of iron atoms are electrochemically active, with the C-coordinated iron being reduced first.^28,29^ Recently, the different stages of sodiation in Prussian Blue compounds are named after their characteristic color, meaning the fully desodiated, half sodiated and fully sodiated compounds are denoted as Prussian Yellow, Prussian Blue and Prussian White, respectively. Still, the site occupancy statistics and resulting atomic structures during sodium (de)­insertion and phase transition to the distorted phase of Na_*x*_Fe­[Fe­(CN)_6_] during battery charging remain elusive. This understanding is critical to making Prussian Blue a competitive commercial electrode material. The sodium orderings, which occur during reversible sodium (de)­intercalation, may be influenced by the known charge ordering at the transition metal sites, although the exact mechanism remains unclear. The Na/vacancy orderings might determine the degrees of distortion as well as the stability of specific Na_*x*_Fe­[Fe­(CN)_6_] phases during sodium (de)­intercalation, which is the primary focus of the current research.

In this study, we investigated the sodium site occupancies in the Na_*x*_Fe­[Fe­(CN)_6_] Prussian Blue framework as the sodium composition (0 ≤ *x* ≤ 2) is varied. This is achieved by a convex hull scheme based on density functional theory (DFT). We study the relevant sodium site occupancies and magnetization as a function of sodium concentration. Our theoretical investigation reveals the tetragonal Na/vacancy ordering in the thermodynamically stable Na_1_Fe­[Fe­(CN)_6_] phase, a feature previously reported in the literature.^30^ Furthermore, as recent studies have confirmed, the rhombohedral distorted phase is more stable than the cubic one for higher concentrations of sodium.^25,31^ Notably, this is the first time that the compositional phase diagram of a Prussian Blue electrode has been derived from first-principles for all 24 of the 24d sites. Our results provide valuable insights into the complexities of sodium (de)­insertion in Na_*x*_Fe­[Fe­(CN)_6_] electrodes, which are crucial for the further improvement of high-rate capacity Prussian Blue electrodes.

## Methods

The DFT^32–34^ calculations were performed, using version 6.2.1 of the plane-wave method based code Vienna Ab-initio Simulation Package (VASP).^35,36^ The projector augmented wave (PAW) approach^36,37^ was utilized to replace the core electrons. To address the known issue of overlocalization, the PBE functional^38^ was employed together with Dudarev’s version of the Hubbard *U*-correction,^39^ which was applied to the iron *d*-states. The *U* values were adapted from previous studies,^31,40^ which indicated that PBAs are best represented with distinct *U*-values for each iron site. In this case, a Hubbard correction of 7 eV was assigned to the *d*-states of the high-spin iron within the FeN_6_ octahedra, while a value of 3 eV was used for the *d*-states of the low-spin iron with the FeC_6_ octahedra. Atomic structures were optimized until the energy difference in the electronic self-consistent field (SCF) fell lower than 10^–6^ eV, and a force convergence criteria of 10^–2^ eV/Å was employed. Furthermore, all calculations used a γ-centered *k*-point grid of 5 × 5 × 5 for cubic structures and 5 × 5 × 3 for rhombohedral ones. The plane-wave cutoff was set to 550 eV and Gaussian smearing with a width of 0.1 eV was used. Additionally, the total excess majority spin electrons were held constant to ensure convergence to the correct high-spin-low-spin spin state on the iron atoms.

In order to explore a sufficiently large number of sodium orderings in cubic Na_*x*_Fe­[Fe­(CN)_6_] Prussian Blue, we enumerated all symmetrically distinct configurations within the conventional unit cell containing up to 4 formula units (56 atoms for *x* = 0) with a resolution on the composition axis of Δ*x* = 0.25. All cubic crystal structures are based on entry COD ID 4100931 from the Crystallography Open Database.^41–43^ The initial structure for the rhombohedral modification of Prussian Blue, exhibiting *R*3̅ symmetry and containing three formula units (48 atoms for *x* = 0) was adapted from the structural parameters published by Wang et al.^27^ The resolution for the composition axis is given as Δ*x* = 0.33 due to the change in unit cell size. The enumeration was performed using the Python Materials Genomics (pymatgen) package.^44^ For each composition, we selected a maximum of 60 structures with the lowest Ewald energy,^45,46^ calculated using point charges (Na = +1, N = −3, C = +2, and Fe = +2–3 depending on the sodiation states) to ensure computational feasibility. In total, we performed DFT structure relaxations (including relaxation of cell volume, cell shape and ionic positions) for 347 structures across the compositional range 0 ≤ *x* ≤ 2.

The convex hull was generated by calculating the mixing energies Δ*E*_mix_ per formula unit (f.u.) for following equation

1 $$\Delta E_{mix}=E\left({\mathrm{Na}}_{x}\mathrm{FeFe}{\left(\mathrm{CN}\right)}_{6}\right)-\left[\frac{x}{2}E\left({\mathrm{Na}}_{2}\mathrm{FeFe}{\left(\mathrm{CN}\right)}_{6}\right)+\left(1-\frac{x}{2}\right)E\left(\mathrm{FeFe}{\left(\mathrm{CN}\right)}_{6}\right)\right]$$

where *E*(Na_*x*_FeFe­(CN)_6_) represent the total energy per f.u. for a system with *x* intercalated sodium atoms. *E*(Na_2_FeFe­(CN)_6_) and *E*(FeFe­(CN)_6_) are the reference energies per f.u. for the completely sodiated and desodiated systems, respectively. The molar fraction of sodium intercalated in the material is represented by $\frac{x}{2}$ .

## Results and Discussion

### Applied Workflow

The schematic representation of the workflow is intended to aid in conveying the logical flow among the different stages of our research process. Depicted in Figure 1, it visually outlines the sequential order of tasks in a flow-diagram format.

**Figure 1:**
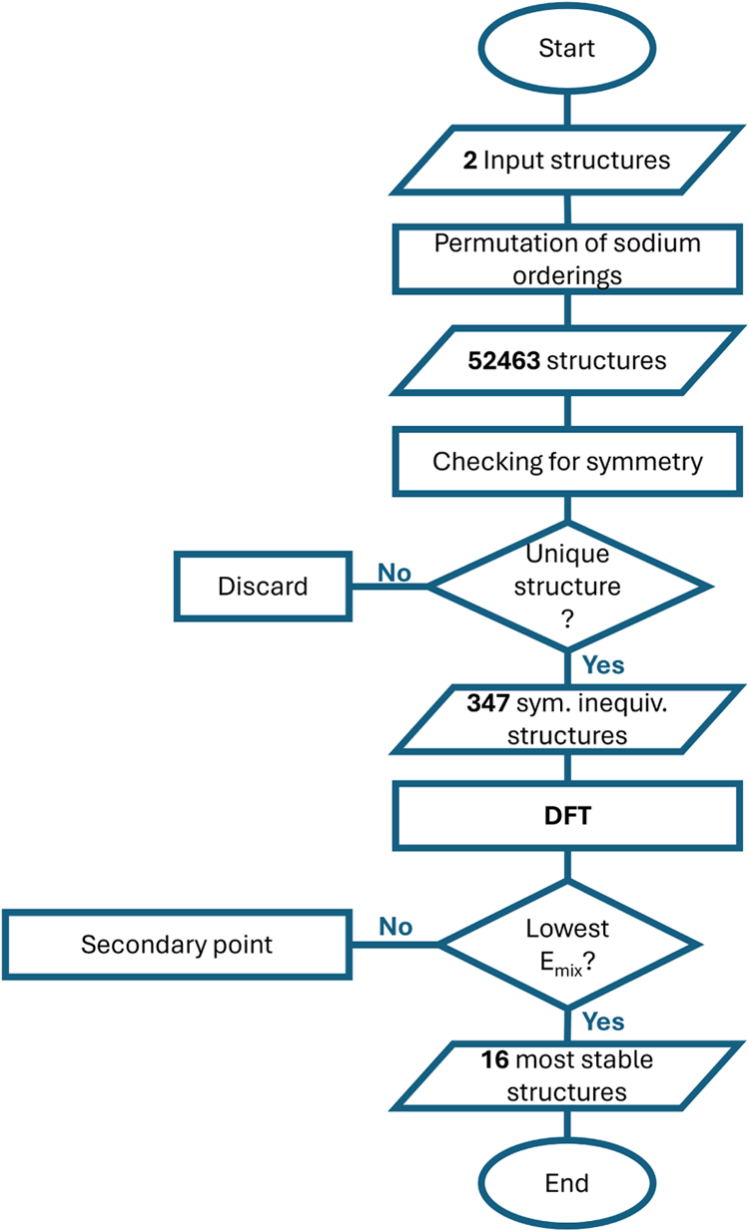
Schematic representation of the workflow used in this study, provided to illustrate the sequence of tasks in a flow-diagram format. Ovals are used to signify a start/end-node, parallelograms denote data input/output nodes, squares indicate a task given to the machine and diamond shapes represent conditional nodes.

The workflow begins with the selection of the two input structures: one representing the cubic modification of Prussian Blue and the other representing the rhombohedral modification. Following the identification of all sodium sites within these structures, the pymatgen library is used to generate permutations of possible sodium orderings for each concentration. Each generated structure is checked to determine if it matches any previously generated, symmetrically equivalent structure. If a duplicate is found, the new structure is discarded; otherwise, it is retained. This iterative process continues until either all permutations are examined or at least 60 symmetrically unique structures are identified for each sodium concentration. In this study, a total of 52,463 structures were generated, of which 347 were confirmed to be symmetrically unique and subsequently evaluated using DFT. For each sodium concentration, the structure with the lowest calculated mixing energy is specifically highlighted in Figures 3 and 7, while the other configurations are represented as light-gray secondary points within the convex hull plots. Upon identifying the most energetically stable sodium configurations, the workflow concludes.

Throughout the application of this workflow, we observed that the Ewald summation method aligns closely with DFT results for structures of high sodium concentrations (*x* ≥ 1), while structures with lower sodium content displayed a weaker correlation. This discrepancy might arise because, in high-concentration systems, the sodium interaction energy contributes significantly to the total energy, whereas, in low-concentration structures, the sodium atoms are more strongly shielded by the framework and separated by larger distances. Although this initially seems to present a challenge for screening low-sodium configurations, it actually does not impact the prescreening process, as these structures have fewer possible configurations. Consequently, all potential sodium orderings are generated, and all symmetrically unique configurations are included in the analysis. The value of prescreening becomes especially evident when approaching the threshold of 60 symmetrically unique configurations, at which it starts to become computationally expensive to include all possible configurations in the DFT calculations. At this stage, where it is crucial to ensure that the lowest energy configuration is included, the Ewald summation method is most accurate. Although there is a minimal risk of excluding critical configurations, the computational resource savings offered by the prescreening justify the trade-off.

### Current Approach and Its Challenges

Currently, two distinct intercalation models are used for the simulation of Prussian Blue (analogue) structures within theoretical studies. We shall explore these models using the example of a fully sodiated Prussian White crystal structure. Either the charge carrier atoms are placed into the 8c position according to Wyckoff,^28,29,47,48^ or shifted by a uniform vector of a quarter of the conventional unit cell along one of the three spatial axis to occupy the 24d position.^28–31^ Although it is well-established that small cations, such as sodium, prefer to occupy the 24d sites,^28^ the unit cell remains cubic only when these cations are positioned within the large voids at the 8c sites. The calculation of the sodium intercalation process into the 24d position without any restrictions on the degrees of freedom allows for a more accurate energetic description. However, the resulting distortion of the unit cell contradicts the experimental findings of the cubic symmetry.^25,30^ This discrepancy is attributed to the sodium-induced expansion of the Fe–CN–Fe squares within their plane, which influences two spatial dimensions while leaving the third axis unaffected. Although the sodium ions are arranged uniformly and periodically to minimize the energetics, the distribution of sodium across all available sites remains highly inhomogeneous.

Moreover, the current approach for sodium distribution across the available sites does not result in the most stable configuration, as determined by a simple electrostatic screening using the Ewald summation method. The most stable configuration is achieved by rotating all the sodium ions in one-half of the unit cell by 90° around a neighboring cyanide ligand, further minimizing sodium–sodium interactions. This behavior can be understood by examining the distances between neighboring atoms: in the current configuration, the distance between sodium atoms is about 5.2 Å, while rotating the sodium atoms increases the distance between the rotated and nonrotated atoms to 6.4 Å. The intrahalf distances remain unchanged, resulting in a net increase in sodium–sodium distances. A comparison between the conventional sodium distribution model in Prussian White and the electrostatically preferred arrangement is provided in Figure 2.

**Figure 2:**
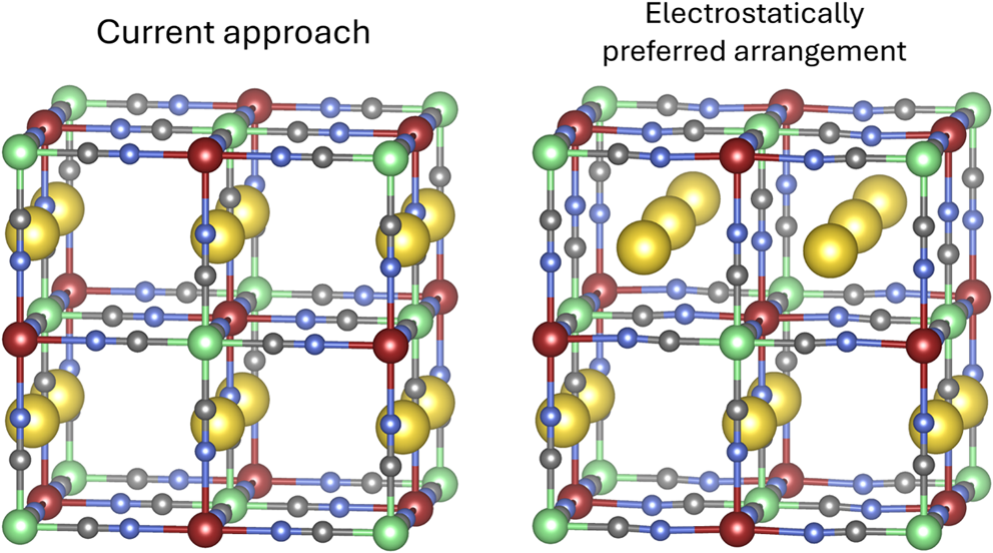
Depiction of the current approach to model the sodium distribution in Prussian White (left) and the preferred arrangement found by an electrostatic screening of the sodium arrangements via the Ewald summation method. High- and low-spin iron atoms are represented in red and green, respectively. Carbon atoms are shown in gray, nitrogen atoms in blue, and sodium atoms in yellow.

Proposing this new sodium arrangement within the fully sodiated cubic Prussian Blue compound is crucial, as the reduced sodium–sodium interactions enhance the accuracy and mitigate distortions that arise when structural optimizations are performed without constraints on the degrees of freedom. This finding is important for future experimental studies and in particular for future theoretical studies that calculate the phase diagram, simulate spectra, compare the relative stability of the rhombohedral and cubic modification or employ molecular dynamics simulations aimed at capturing the sodium diffusion mechanism within the system.

### Extended Convex Hull

To investigate the importance of the exact sodium distribution within the Prussian Blue framework, we calculated the full convex hull of up to 8 sodium ions being distributed over all 24 available 24d sites within the conventional unit cell. The resulting plot is shown in Figure 3. Aside from the fully desodiated and sodiated structures, the convex hull shows only one stable intermediate concentration of sodium, at exactly one sodium per formula unit. The stability of this intermediate is expected, as it closely resembles the original Prussian Blue compound^49,50^ with the only modification being the substitution of potassium for sodium.

**Figure 3:**
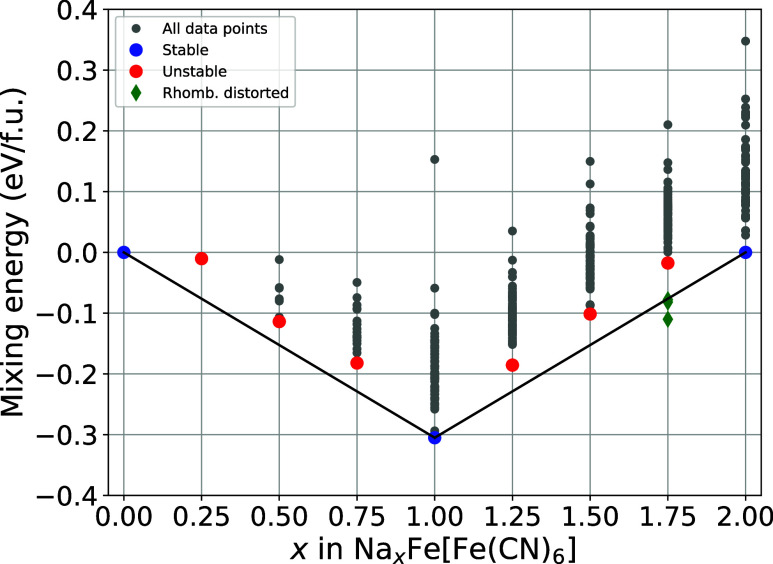
Convex hull plot of the cubic Prussian Blue system. Plotted are the mixing energies per formula unit vs various sodium concentrations. The most stable compound for each concentration of sodium is highlighted—stable ones in blue, unstable in red. Geometries that distorted rhombohedrally are represented by green diamonds.

Three of the generated structures for *x* = 1.75 in Na*_x_* Fe­[Fe­(CN)_6_] (Na_1.75_FeHCF), indicated by green diamonds, were found to be no longer cubic and distorted after relaxation, with energies below the convex hull. However, due to the rhombohedral-like configurations, these structures were excluded from the cubic convex hull and are still shown in Figure 3 for completeness. Further details, including the geometries of each point below the hull, are provided in the Supporting Information (SI), Section 1—“Rhombohedral distorted geometries”. It should be noted that the rhombohedral modification of Prussian Blue is favored at sodium concentrations exceeding one sodium per formula unit.^27,30,51^ While this effectively validates the accuracy of the computational framework, the primary focus of this study is the distribution of sodium ions within the cubic crystal structure across the entire range of sodium concentrations.

Upon evaluation of all identified metastable configurations, no mixing energy difference greater than ∼25 meV was observed between the most stable geometry and the next metastable configuration of the same sodium concentration. A pronounced clustering of configurations in terms of energy is observed across all sodium concentrations, indicating a strong degeneration of site occupations within the same sodium concentration in the Prussian Blue framework. To elucidate the effect of temperature, the average kinetic energy of the charge carriers at room temperature is estimated via the Boltzmann law. This results in 3/2 × *k*_B_ × 298 K = 0.03853 eV ≈ 39 meV, that still neglects the effects of (locally) elevated temperatures during battery operation, as well as the added driving force due to the nonequilibrium nature of the system under operating conditions. Comparing the spread in the energies of different sodium arrangements within the same sodium concentration of 25 meV with the thermal energy of about 39 meV per atom at room temperature, it can be assumed that most of the sodium distribution patterns are degenerate during battery applications. Moreover, the presence of numerous energetically degenerate structures provides further evidence for the experimentally well-established high mobility of charge carriers within the Prussian Blue framework. While this does not directly estimate the energy barrier between these configurations, having a multitude of available sites for charge carriers normally facilitates mobility. Additionally, the lack of any significant long-range order—except for the tetragonal Prussian Blue arrangement—implies that no particular order is stabilized. As such, no additional energy contribution is imposed on the migration barrier for charge carriers to escape this kind of long-range order.

The most stable geometry for each concentration of sodium is shown in Figures 4 and 5. The geometry of the fully desodiated cell is omitted in the main text and can be viewed in the Supporting Information in Figure S2. In the lowest calculated sodium concentration, all 24d positions are still degenerate, hence only one configuration is calculated (Na_0.25_FeHCF).

**Figure 4:**
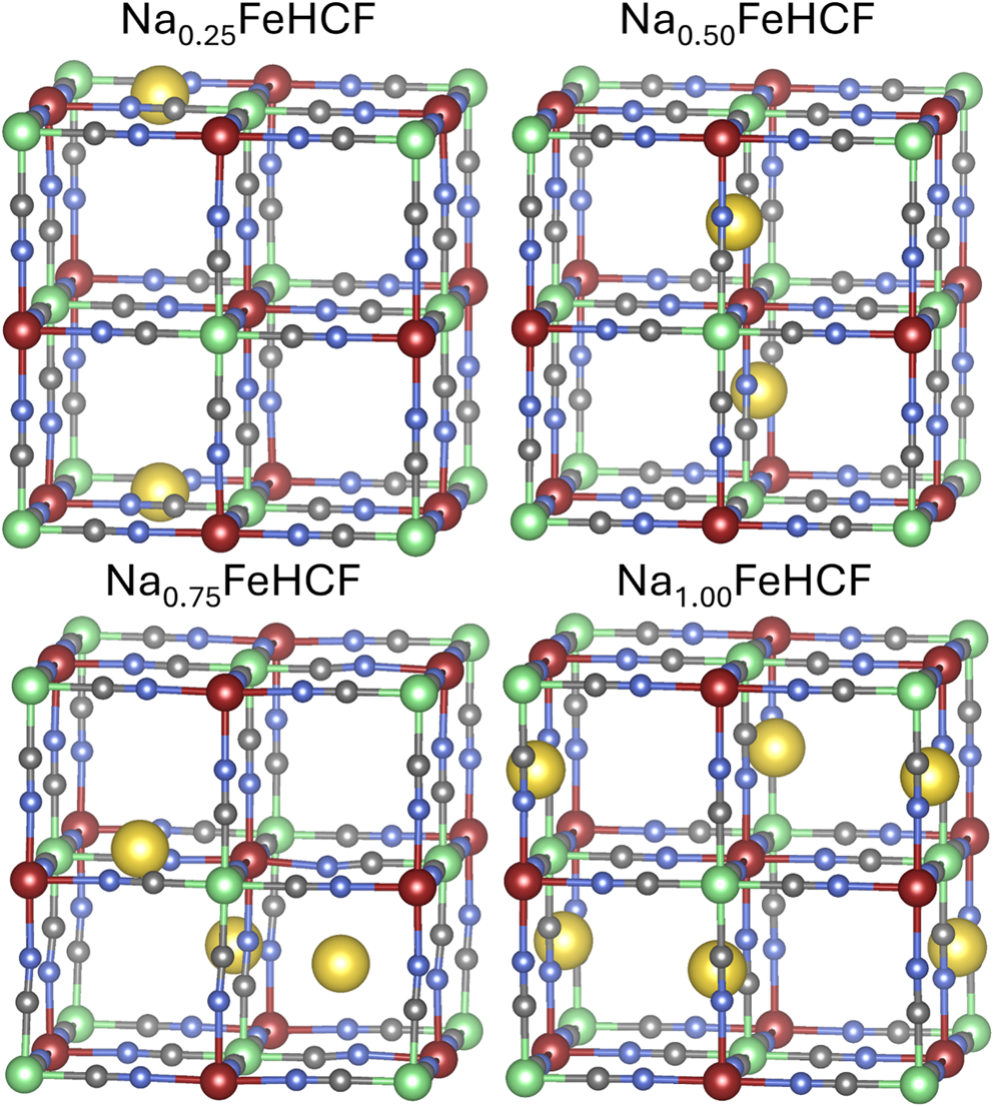
Depiction of the most stable structures for the sodium concentrations Na_0.25_ to Na_1_ per f.u.. High- and low-spin iron atoms are represented in red and green, respectively. Carbon atoms are shown in gray, nitrogen atoms in blue, and sodium atoms in yellow.

**Figure 5:**
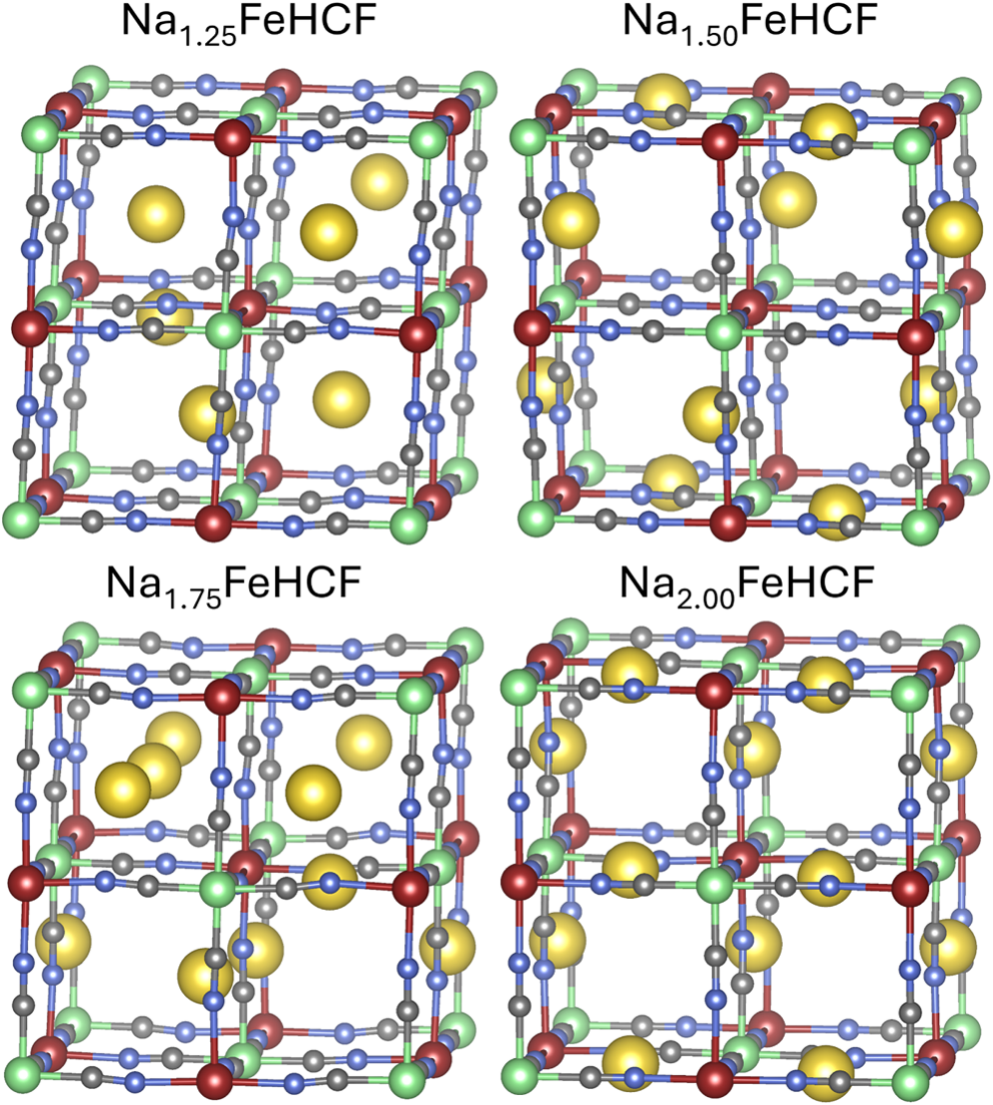
Depiction of the most stable structures for the sodium concentrations Na_1.25_ to Na_2_ per f.u.. High- and low-spin iron atoms are represented in red and green, respectively. Carbon atoms are shown in gray, nitrogen atoms in blue, and sodium atoms in yellow.

Adding another sodium atom into the conventional unit cell, the concentration Na_0.5_FeHCF is reached. The sodium atoms are arranged in a diagonal stack within a *yz*-plane, leading to alternating layers of empty and diagonally stacked *yz*-planes throughout the crystal. This would, of course, also apply to any symmetry-equivalent structures, where other planes are stacked on top of each other. The results published by Wang et al.^30^ align with this finding. The geometry found at this concentration already deviates from the current approach. This could be achieved by shifting any of the sodium atoms by half a unit cell parallel to their plane.

Adding more sodium into the system, the sodium configuration in the lowest energy structure for Na_0.75_FeHCF does not exhibit any noticeable long-range patterns. The structure of Na_1_FeHCF equals the structure that is achieved by minimizing sodium–sodium interactions. The tetrahedral arrangement of the sodium atoms in the half-filled compound is shown to be an especially stable structure. However, the structures obtained in our study show a slight shift of the sodium atoms out of the Fe–CN–Fe plane, optimizing their interactions further.

The next higher concentration of sodium, Na_1.25_FeHCF, does not exhibit any significant long-range ordering. However, it should be noted that this structure deviates distinctly from the tetragonal sodium atom configuration in Prussian Blue, in which an additional sodium atom was simply inserted.

Na_1.5_FeHCF shows the same diagonal arrangement throughout the cell that we observed to be favored in the Na_0.5_ concentration. The sodium arrangement in Na_1.75_FeHCF, on the other hand, seems to be evenly distributed between all planes, reducing distortions.

In the fully intercalated framework, at a concentration of Na_2_FeHCF, the configuration exhibits alternating layers of sodium stacks along the *z*-direction, followed by stacks of sodium oriented along the *x*-direction. This configuration is the same as the one identified as the most favorable arrangement in the Ewald summation screening.

Overall, no clearly favored arrangement of sodium can be found for any of the intermediate concentrations, except for the tetragonal configuration of sodium atoms in Prussian Blue. This finding contradicts the common approach of sequentially occupying the sodium positions associated with Prussian Blue. Rather the positions appear to be strongly degenerate for all intermediate concentrations that do not lie on the convex hull.

In Table 1, the most stable structures are further compared with regard to their lattice constant and angles, unit cell volume, averaged magnetization on the iron center, and the calculated mixing energy per formula unit. As seen in earlier studies,^29,30,51^ the calculated lattice constants and the unit cell volume do not reproduce experimental findings by not significantly changing from the fully desodiated state to the half-sodiated state. In the calculations, the electrostatic attraction between the intercalated charge carriers and the framework is equal to the volume expansion caused by the increased occupancy of the 24d site. Going from Prussian Blue to Prussian White, the lattice constants then expand by about 2.2%.

**Table 1:** Structural Properties, Magnetization and Mixing Energies of the Most Stable Prussian Blue Structure for Each Concentration of Intercalated Sodium

property	Na_0_	Na_0.25_	Na_0.5_	Na_0.75_	Na_1_	Na_1.25_	Na_1.5_	Na_1.75_	Na_2_
lattice constant [Å]									
*a*	10.320	10.325	10.298	10.291	10.239	10.344	10.368	10.444	10.502
*b*	10.320	10.293	10.315	10.299	10.320	10.345	10.379	10.475	10.503
*c*	10.320	10.325	10.315	10.291	10.320	10.310	10.399	10.441	10.570
volume [Å^3^]	1100.2	1096.9	1095.3	1090.5	1088.9	1101.3	1114.9	1141.4	1166.0
angles [°]									
α	89.7	90.0	91.8	90.8	87.0	88.9	85.8	88.7	90.0
β	89.7	88.5	90.0	89.2	90.0	91.1	87.4	91.3	89.9
γ	90.1	90.0	90.0	89.2	90.0	87.0	90.5	88.7	90.1
μ_Fe^LS^‑C_ [μ_B_]	1.02	0.85	0.58	0.37	0.14	0.12	0.09	0.06	0.03
μ_Fe^HS^‑N_ [μ_B_]	4.33	4.31	4.32	4.31	4.31	4.14	3.97	3.81	3.65
Δ*E*_mix_ [eV/f.u.]	0	–0.010	–0.114	–0.182	–0.305	–0.186	–0.101	–0.018	0

The overall volume change from Prussian Yellow to Prussian White would amount to a 6% expansion. A plot of the volume in dependence on the concentration of sodium is presented in the SI in Figure S3. The observed changes in the lattice constants and cell volume are in agreement with the experimental observations by Wu et al.^52^ They obtained a change in the lattice constant from 10.18 to 10.41 Å (2.2%), which results in a volume expansion of 6.9%. Nevertheless, the complete attribution of the volume expansion to the step from Prussian Blue (Na_1_FeHCF) to Prussian White (Na_2_FeHCF) is most likely off, as the experiments show a linear increase in the lattice constant. Our calculations show that the angles of all unit cells distort slightly from the cubic symmetry because of the asymmetric distribution of the sodium atoms. Still, the distortions are small, mostly being kept below 2 °deviation from the original 90 °value.

The mixing energies presented in Table 1 serve as an additional representation of the most stable geometries illustrated in Figure 3, providing precise numerical values for these geometries. It should be noted, that we observe the mixing energies to be almost perfectly mirrored at the Prussian Blue structure (*x* = 1). This mirrored trend indicates that the concentration difference to the Prussian Blue structure might be a good indicator for the structural stability of these systems.

The values for the average magnetization of the Fe–N_6_ and Fe–C_6_ centers show the selective oxidation of the iron centers during the sodiation of the material. The change in magnetization is further presented in Figure 6. The full process has been reported in detail in an earlier publication.^31^ To summarize, as the carbon coordinated, low-spin iron center is oxidized first, the average magnetization decreases linearly from 1.02 μ_B_ to 0.14 μ_B_ during the first half of the sodiation process. The remaining electron density can be explained by a delocalization effect from the neighboring Fe–N_6_ octahedra. The magnetization decreases further to almost zero during the second half of the sodiation process. In contrast, the magnetization of the nitrogen-coordinated, high-spin iron atoms does not significantly change during the intercalation of the first sodium per formula unit (f.u.). During the intercalation of the second sodium ion, the magnetization drops from 4.3 μ_B_ to 3.65 μ_B_ in the fully sodiated compound. The values for the high-spin iron are lower than the formally expected number of unpaired electrons due to overdelocalization, a phenomenon already well-known in the type of functional used in this study.

**Figure 6:**
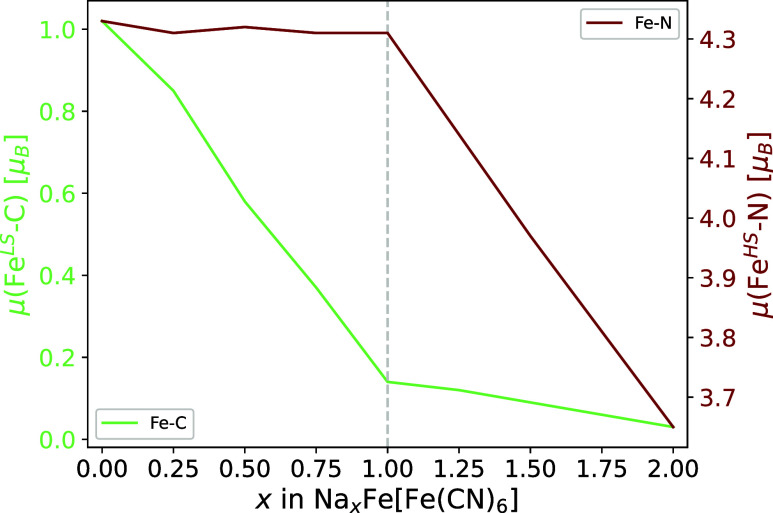
Plot of the selective oxidation behavior represented by the magnetic moments of the carbon-coordinated, low-spin iron center μ­(Fe^LS^-C) in green and the nitrogen-coordinated, high-spin iron center μ­(Fe^HS^-N) in red.

### Rhombohedral PB and the Convex Hull

While this work focuses on the cubic modification of Prussian Blue with its highly flexible sodium arrangements, we also include the convex hull of the rhombohedral modification to aid in the comparison. Figure 7 presents the convex hull of the Prussian Blue material, including the results for the mixing energy per formula unit for the rhombohedral modification, overlaid with the previous results.

**Figure 7:**
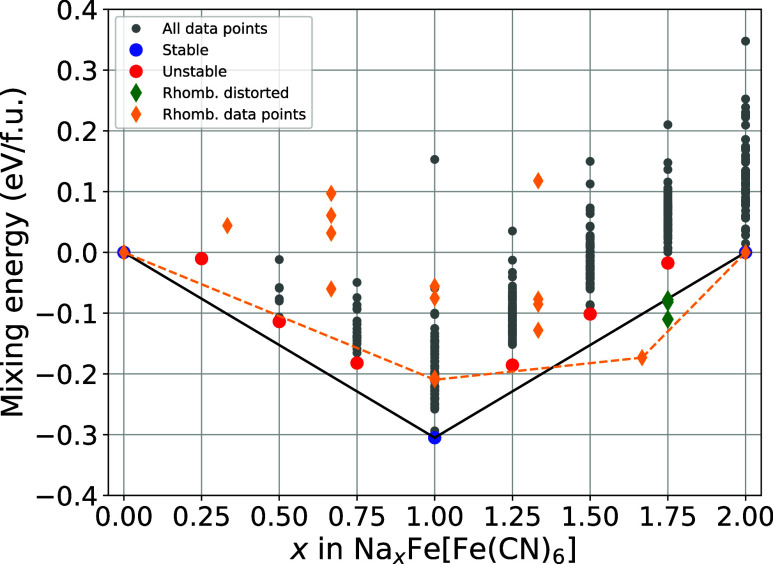
Convex hull plot of the cubic and rhombohedral Prussian Blue system. Plotted are the different concentrations of sodium vs the mixing energies per formula unit. The most stable compounds for each concentration of sodium in the cubic system are highlighted—stable ones in blue, and unstable ones in red. Geometries that distorted rhombohedrally within the cubic unit cell are represented by green diamonds. The separately calculated convex-hull of the rhombohedral system is shown by orange diamonds. Additionally included is a theoretical hull only comprised of the rhombohedral points, represented by an orange, dashed line.

In accordance with well-established knowledge, no stable structures of the rhombohedral modification can be found at or below the strongly stabilized half-sodiated Na_1_FeHCF structure. The structure for Na_1.33_FeHCF does not agree with current findings in the literature,^12,25^ as it is not stabilized compared to the cubic modification. On the other hand, the structures of Na_1.66_FeHCF are well aligned with the stabilization of the rhombohedral modification above one sodium per formula unit. A possible reason for the deviation is the uniform *U* value applied across the entire range of sodium concentrations by the PBE + *U* functional. This may lead to an overestimation of the thermodynamic stability of the cubic phase relative to the rhombohedral one. The rhombohedrally distorted structures generated from the cubic modification at Na_1.75_FeHCF fit well into the new convex hull, displaying metastability when compared with the rhombohedral phase.

Analyzing the theoretical convex hull based solely on the rhombohedral data points, represented by the orange dashed line in Figure 7, reveals stable structures at Na_1_FeHCF and Na_1.66_FeHCF. It appears that even in the rhombohedral system—where the tetragonal sodium arrangement is replaced by layers of sodium perpendicular to the *c*-axis—the alternating arrangement of Fe^2+^–C and Fe^3+^–N in Na_1_FeHCF remains energetically favorable. The sodium layers prefer to be half filled rather than alternating between filled and unfilled. A geometrical description of these systems can be found in the SI, Section 4—“Rhombohedral Geometries on the Hull”. This rhombohedral exclusive convex hull would intersect the purely cubic convex hull at about 1.4 sodium per f.u., marking the transition from the cubic to the rhombohedral system.

### Reliability of PBE + *U*

To assess the reliability of our Hubbard *U*-corrected results, we incorporated HSE06-calculated data points for the cubic modification of Prussian Blue into the convex hull, as shown in Figure 8. While we also attempted to perform HSE06 calculations for the rhombohedral modification, severe convergence issues prevented us from obtaining meaningful results. The HSE06-calculated data follow a similar energy trend to that obtained with PBE + *U* but exhibit a steeper slope in the convex hull. This increased slope suggests that HSE06 provides a more accurate representation of sodium intercalation energetics in Na_*x*_Fe­[Fe­(CN)_6_], likely due to the inclusion of exact exchange in the hybrid functional. The steeper convex hull slope reflects a stronger driving force for ion insertion and improved energetics. While both functionals yield comparable voltage profiles, HSE06 offers a more refined description of the electronic structure, particularly at higher sodium concentrations, where sodium–sodium interactions become increasingly complex.

**Figure 8:**
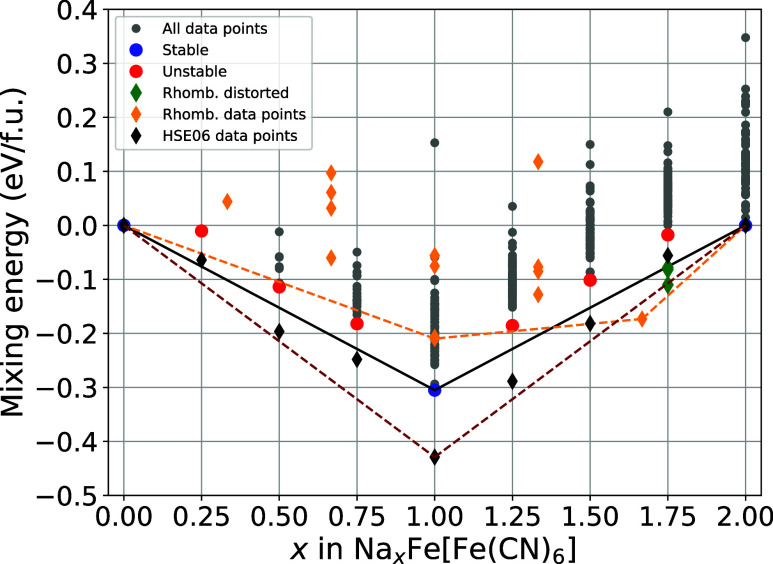
Convex hull plot of the cubic and rhombohedral Prussian Blue system. Plotted are the different concentrations of sodium vs the mixing energies per formula unit. The most stable compounds for each concentration of sodium in the cubic system are highlighted—stable ones in blue, and unstable ones in red. Geometries that distorted rhombohedrally within the cubic unit cell are represented by green diamonds. The separately calculated convex-hull of the rhombohedral system is shown by orange diamonds. Additionally included is a theoretical hull only comprised of the rhombohedral points, represented by an orange, dashed line. The added data points obtained by HSE06 hybrid calculations are shown in black and are supplemented by a dashed line to emphasize the improved convex hull.

The voltage curve for the cubic modification of Prussian Blue was calculated using the HSE06 hybrid functional, which incorporates a portion of the exact exchange to improve the accuracy of electronic structure predictions, especially for systems with strong electron correlation. The resulting curve, shown in Figure 9, displays two distinct voltage plateaus, accurately reflecting the experimentally observed electrochemical behavior of the material during sodium-ion intercalation. Within the lower sodium concentrations (0 ≤ *x* ≤ 1) the voltage remains stable at 3.10 V, indicating a region of low energy cost for sodium insertion. This is followed by a drop to 2.24 V for the higher sodium concentrations (1 ≤ *x* ≤ 2). While the calculated voltage for the first plateau is an acceptable estimation of the experimentally measured voltages of ∼3.4 V, the voltage drop of 0.76 V toward the second voltage plateau is significantly stronger than any experimentally observed voltage drops. This discrepancy likely stems from the assumption of a purely cubic phase, necessitated by convergence issues in hybrid functional calculations for the rhombohedral phase. As the rhombohedral phase is energetically preferred in this concentration range, its inclusion would likely increase the second voltage plateau. As such, a more detailed functional assessment for high sodium concentrations in Prussian Blue are reserved for a future study.

**Figure 9:**
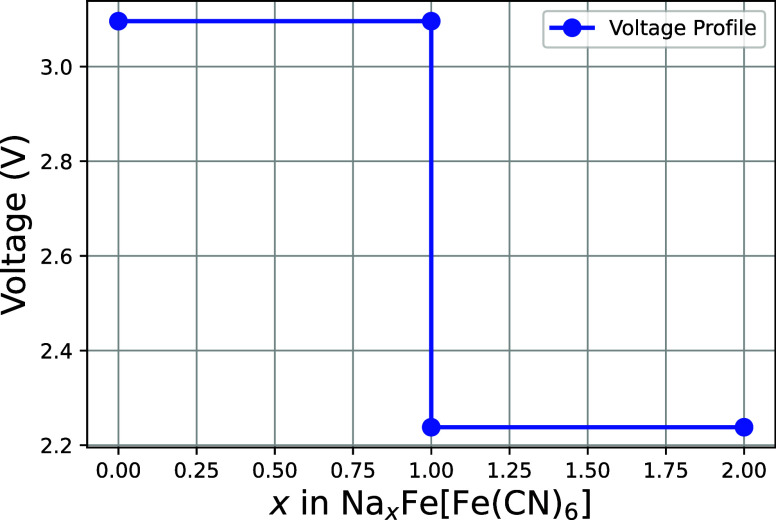
Calculated OCV for the cubic modification of Prussian Blue as calculated from the HSE06 data points. Plotted is the Voltage vs the sodium concentration *x*.

## Conclusions

In this study, we explored the influence of sodium configurations and site occupancy statistics within the cubic phase of Prussian Blue as a cathode material. Initially, we screened all potential sodium arrangements, focusing on symmetry-inequivalent structures, which were subsequently analyzed using density functional theory (DFT). This led to the identification of a novel, more stable sodium arrangement for the cubic Prussian Blue structure, which was validated through further DFT calculations. Notably, we found that purely electrostatic models (Ewald summation) fail to accurately represent sodium–sodium interactions within the cubic framework.

We also introduced the first comprehensive convex hull for the cubic Prussian Blue system, considering all 24 available 24d sites. From this convex hull, we determined an upper bound of 25 meV for the mixing energy difference between the most stable sodium configuration and the next metastable arrangement at equivalent sodium concentrations. A significant clustering of configurations was observed across all sodium concentrations in terms of mixing energy, suggesting considerable degeneracy in sodium configurations and weak sodium–sodium interactions, even between sodium atoms occupying adjacent 24d sites. The convex hull revealed only one stable intermediate sodium concentration at *x* = 1, consistent with two voltage plateaus in the open-circuit voltage (OCV), as observed experimentally.

Our analysis did not identify any stable sodium configuration apart from the tetragonal arrangement in Prussian Blue, implying that the experimentally observed cubic structure likely results from large-scale averaging of multiple sodium arrangements. The mixing energies for the most stable sodium configurations at each concentration are mirrored at the energy profile of the stable Prussian Blue configuration (*x* = 1). Finally, we compared the convex hull of the cubic phase to that of the rhombohedral phase, showing a qualitative agreement with experimental data, although quantitative discrepancies remain. These findings provide enhanced insights into the sodium intercalation mechanism in Prussian Blue materials, and the proposed sodium arrangement in Prussian White might enable more accurate atomistic modeling in future studies.

## Supplementary Material



## Data Availability

The data that support the findings of this study are available from the NOMAD database at https://www.doi.org/10.17172/NOMAD/2025.04.04-1.

## Notes

Additional information on atomic structures and supporting graphs (PDF)

## Notes

The authors have cited additional references within the Supporting Information.^53–60^
