# Supplementary material for: Data-Driven Site Occupancy Statistics in Cubic Prussian Blue
Source: ACS Phys Chem Au. 2025 Apr 8;5(4):346–55. doi: 10.1021/acsphyschemau.5c00001 (PMC12291116; doi:10.1021/acsphyschemau.5c00001)
Supplement: Supplementary file 1 [file pg5c00001_si_001.pdf]

# Data-driven site occupancy statistics in cubic Prussian Blue

Sebastian Baumgart[1], Axel Groß[1,2], and Mohsen Sotoudeh[1]

[1] *Institute of Theoretical Chemistry, Ulm University, Oberberghof 7, 89081 Ulm, Germany*
[2] *Helmholtz Institute Ulm (HIU) for Electrochemical Energy Storage, Helmholtzstraße 11, 89081 Ulm, Germany*

Email: sebastian.baumgart@uni-ulm.de, mohsen.sotoudeh@uni-ulm.de

April 4, 2025

Table S1: Structural properties, magnetization and mixing energies of the rhombohedrally distorted Prussian Blue structures found for the sodium concentration of $Na_{1.75}$FeHCF.

| Property | $Na_{1.75}$FeHCF - v35 | $Na_{1.75}$FeHCF - v37 | $Na_{1.75}$FeHCF - v38 |
|---|---|---|---|
| lattice constant [Å]: | | | |
| a | 9.842 | 9.828 | 9.872 |
| b | 9.864 | 9.875 | 9.829 |
| c | 9.850 | 9.823 | 9.847 |
| Volume [Å$^3$] | 939.6 | 940.5 | 940.3 |
| angles [°]: | | | |
| $\alpha$ | 83.5 | 84.1 | 84.2 |
| $\beta$ | 83.5 | 84.7 | 96.5 |
| $\gamma$ | 83.8 | 84.5 | 96.0 |
| $\mu_{Fe^{LS}\text{-}C}$ [$\mu_B$] | 0.07 | 0.07 | 0.07 |
| $\mu_{Fe^{HS}\text{-}N}$ [$\mu_B$] | 3.78 | 3.78 | 3.78 |
| $\Delta E_{mix}$ [eV/f.u.] | -0.110 | -0.078 | -0.082 |

# 1 Rhombohedral distorted geometries

Though this study focuses on the sodium site distribution in the cubic modification of Prussian Blue, the data points that were perpetually distorting towards the rhombohedral modification shall not be discarded without note. As already mentioned in the main text, some of the cubic structures that were calculated for the concentration $Na_{1.75}$FeHCF, displayed energies below the hull. These structures are presented in Figure S1. Their labels are related to the actual generation of the structures, where the pymatgen code names the structures according to their ewald summation energy in an ascending order.

All excluded structures show a strong resemblance to each other in virtually every metric. For all structures the cooperative out-of-phase rotation of the $FeC_6$ and $FeN_6$ octahedra in all three directions of space ($a^-a^-a^-$ rotation after Glazer)[1–6] is clearly visible. Such a rotation often causes the trigonal distortion within the octahedra.[7,8] The rotation is accompanied by a shift of the sodium atoms out of the normal 24d / face-centered positions along the [111] axis of the cubic cell towards its vertices that contain $FeN_6$ octahedra. This in turn optimizes the Na-N interactions in the crystal, providing an octahedral coordination of nitrogen around the sodium atoms. Within these Na-N octahedra the sodium ions move towards the face site which is

not face-connected to an $FeC_6$ octahedra as to optimize the space available to them.[9] This distortion within a cubic unit cell is connected with a sharp decrease in volume, as can be seen in Table S1.

The volume is dropping from 1141.4 Å$^3$ of the most stable structure of the $Na_{1.75}$FeHCF concentration to around 940 Å$^3$ (-17.6%). This change is reflected in the decrease of the lattice constants by a corresponding value. The angles of the unit cell are comparably more strongly distorted by about 6° instead of less than 3° for the structures in the main text. The magnetization values, on the other hand, do not differ from the most stable compound for both iron centers.

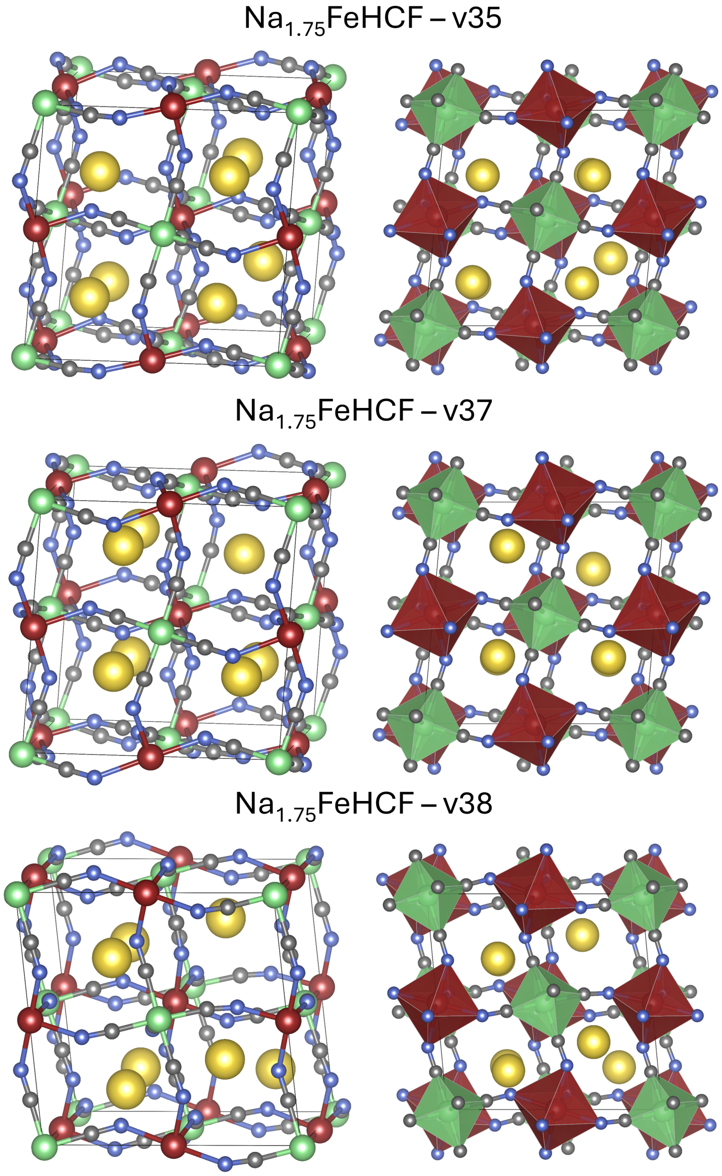


Figure S1: Visualization of the strongly distorted geometries for x = 1.75 in $Na_xFe[Fe(CN)_6]$ that were excluded from the cubic convex hull due to their rhombohedral-like configurations. High- and low-spin iron atoms are represented in red and green, respectively. The coordination polyhedra are colored to match the respective central atom, with their transparency increased to enhance the visibility of the underlying structure. Carbon atoms are shown in grey, nitrogen atoms in blue, and sodium atoms in yellow.

## 2 Prussian Yellow geometry

In Figure S2, we present the the geometry for the fully desodiated Prussian Yellow structure. No defining features or distortions of any form can be found.

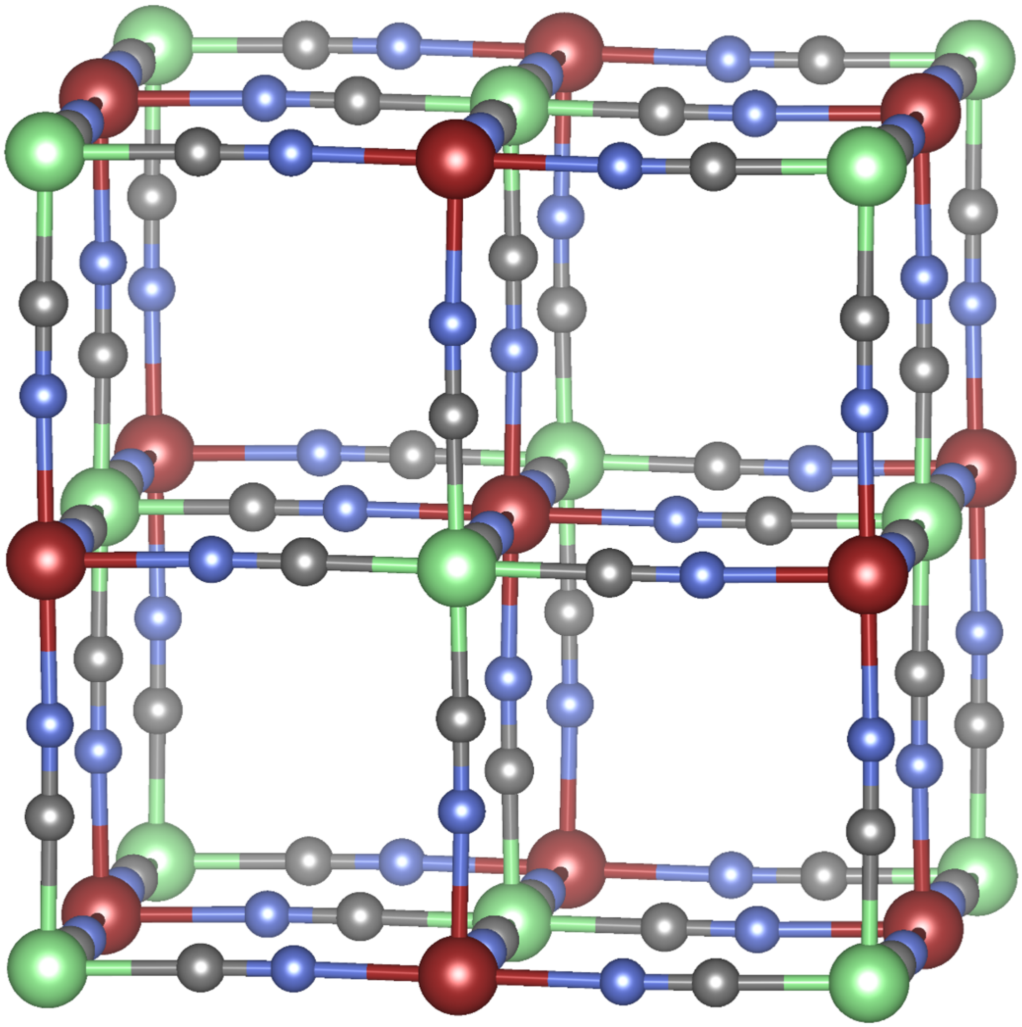

Figure S2: Fully desodiated, Prussian Yellow structure. High- and low-spin iron atoms are represented in red and green, respectively. Carbon atoms are shown in grey, nitrogen atoms in blue, and sodium atoms in yellow.

# 3 Plotted volume change

The plot in Figure S3 shows the change in unit cell volume in dependence on the concentration of sodium per formula unit intercalated into the material. Contrary to experimental results, the volume of the material is decreasing between zero and one sodium per f.u.. Only afterwards the volume is expanding again in an almost linear fashion going towards 2 sodium per f.u.. While the overall expansion from the fully desodiated to the fully sodiated system is matching experimental results, it is save to say that one would expect the volume to rise linearly. It is apparent, that in our calculations, the volume expansion caused by the increased occupancy of the 24d site is competing with the increased electrostatic attraction between the charge carriers and the framework.

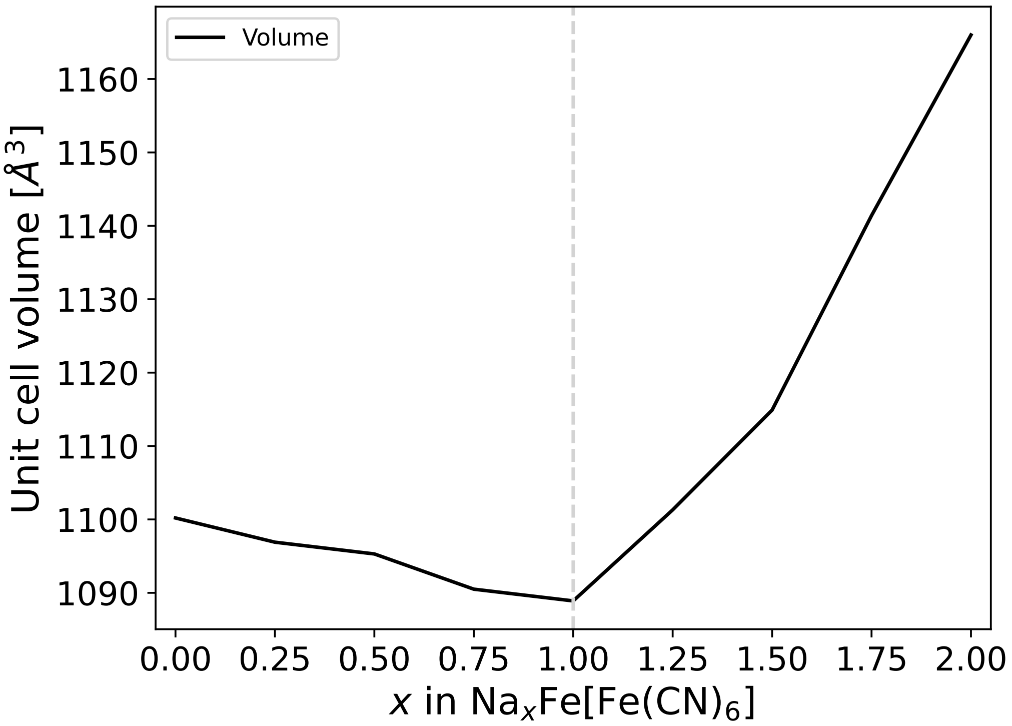


Figure S3: Plot of the unit cell volume versus the concentration of sodium in the Prussian Blue material.

# 4 Rhombohedral geometries on the hull

In this section, we present the structures making up the convex hull of the rhombohedral modification. The configurations depicted in Figure S4 were found to be stable for their respective concentration.

The arrangement at $Na_{1.00}$FeHCF consists of all three layers of sodium being half-filled. The third layer is occupied by a sodium in the middle of the cell, which is mostly obstructed by a $FeN_6$ octahedra in front of it. The configuration for $Na_{1.67}$FeHCF shows a twice filled top layer within the unit cell, while both lower layers are only half filled. The last sodium in this structure moved down from the middle layer into the empty space between the middle and bottom sodium layer during the optimization, as if to participate in the saturation of both. Lastly, the fully sodiated structure shows all sodium layers to be occupied twice.

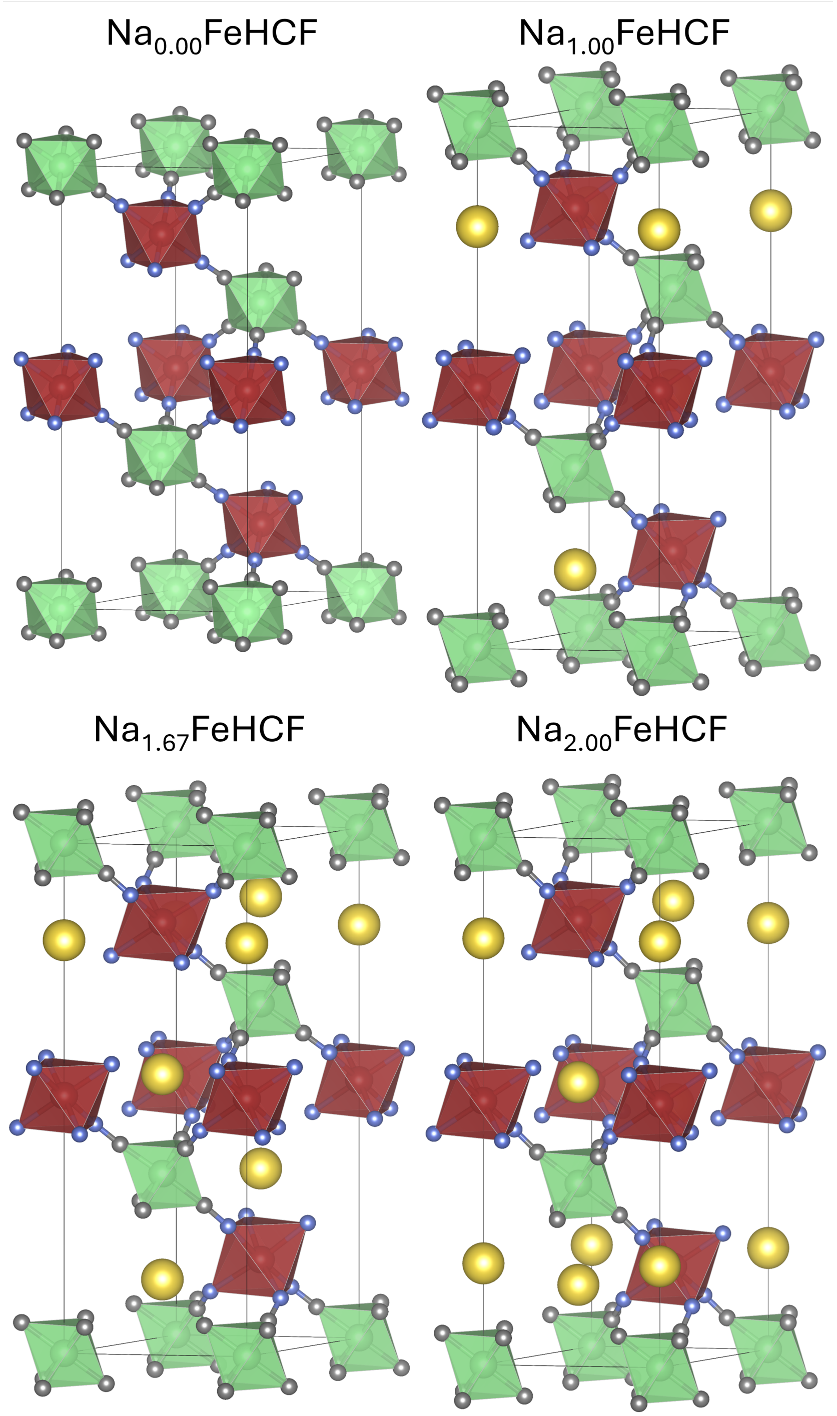


Figure S4: Depiction of the stable geometries on the rhombohedral-only hull. High- and low-spin iron atoms are represented in red and green, respectively. The coordination polyhedra are colored to match the respective central atom, with their transparency increased to enhance the visibility of the underlying structure. Carbon atoms are shown in grey, nitrogen atoms in blue, and sodium atoms in yellow.

# References


[1] A. M. Glazer, *Acta. Crystallogr. B.* **1972**, *28*, 3384–3392.

[2] A. Glazer, *Acta Crystallogr. Sec. A: Crystal Physics Diffraction Theoretical and General Crystallography* **1975**, *31*, 756–762.

[3] C. J. Howard, H. T. Stokes, *Acta. Crystallogr. B. Struct.* **1998**, *54*, 782–789.

[4] C. J. Howard, H. T. Stokes, *Acta. Crystallogr. B. Struct.* **2002**, *58*, 565–565.

[5] C. J. Howard, B. J. Kennedy, P. M. Woodward, *Acta. Crystallogr. B. Struct.* **2003**, *59*, 463–471.

[6] H. L. Boström, W. R. Brant, *J. Mater. Chem. C* **2022**, *10*, 13690–13699.

[7] M. Sotoudeh, M. Dillenz, A. Groß, *Adv. Energy Sustainability Res.* **2021**, *2*, 2100113.

[8] M. Sotoudeh, M. Dillenz, J. Döhn, J. Hansen, S. Dsoke, A. Groß, *Chem. Mater.* **2023**, *35*, 4786–4797.

[9] S. Baumgart, M. Sotoudeh, A. Groß, *Batter. Supercaps* **2023**, *6*, e202300294.
